# Dislocation analysis and structural characteristics of feal alloys under different heating rates using molecular dynamics simulation

**DOI:** 10.1038/s41598-025-93119-w

**Published:** 2025-12-16

**Authors:** Ridwan Ridwan, Sudarno Sudarno, Wahidin Nuriana

**Affiliations:** Department of Mechanical Engineering, Universitas Merdeka Madiun, Madiun, 63133 Indonesia

**Keywords:** Molecular dynamics study, Heating rate, Ferroaluminum, Dislocation analysis, Transition phase, Engineering, Materials science, Atomic and molecular physics

## Abstract

In this study, molecular dynamics simulations are performed to investigate the dislocation behavior and structural characteristics of ferroaluminum (FeAl) alloys under different heating rates. This alloy has a remarkably low cost, is easy to fabricate, and exhibits good corrosion, sulfidation, and oxidation resistance, making it suitable for applications such as furnace fittings, heating elements, and heat exchange pipes. Molecular dynamics simulations are frequently used to analyze the behavior of atoms and molecules based on physical laws, such as classical Newtonian mechanics. During the heating process, the temperature increases from 300 K to 2500 K, exceeding the material’s melting point. The effect of the heating rate varies at 44, 27, 20, and 16 K ps^−1^. The results indicate that higher heating rates cause dislocations to occur at higher temperatures in the bcc crystal structure, whereas lower heating rates result in dislocations at lower temperatures. Additionally, faster heating rates cause phase transitions to take place at higher temperatures compared to slower heating rates, influencing the distribution of local structures during the transition phase. This study primarily aims to enhance the understanding of structural changes in the FeAl alloy at the atomic scale.

## Introduction

Ferroaluminum based on FeAl and Fe_3_Al alloys is notable for its ease of fabrication and excellent resistance to corrosion, sulfidation, and oxidation. These advantages make it suitable for various applications, including furnace fittings, heating elements, heat exchange pipes, automobiles, industrial valve components, sintered porous metal-gas filters, components for molten salt applications, and catalytic converter substrates^1^. During fabrication, FeAl is typically prepared by heating the material until it melts, followed by solidification from the liquid state. This process is of particular interest to researchers because heating can lead to structural variations in the material^2,3^. While most studies focus on its effects on mechanical properties^2,3^, analyses of the heating process’s influence on atomic-scale characteristics remain relatively limited.

Numerous experimental and numerical studies have been conducted to investigate the structural characteristics of the FeAl alloys and Fe- or Al-based materials. Liu et al.^4^ examined the oxidation behavior of both uncoated and coated FeAl materials, indicating that differences in thermal expansion rates between the metal and its oxide lead to stress development at the interface between the substrate and oxide layers. In 2001, Li et al.^5^ utilized molecular dynamics (MD) simulations to analyze clusters within a liquid FeAl alloy. Their study revealed that the different clusters in the liquid state exhibited distinct microstructures and were rearranged in a specific order over time. In 2007, Tang et al.^6^ investigated the influence of harmonics on the B2-FeAl (1 1 0) surface using molecular dynamics simulations. Their study suggested that temperature-dependency layer structure factors indicated no disorder on the B2-FeAl (1 1 0) surface until 1300 K. Additionally, Gao et al.^7^ explored the pore structure of porous ferroaluminum (FeAl) materials under different heating rates. They discovered that variations in heating rate led to distinct formation routes for pore structures. In 2012, Ouyang et al.^8^ examined the thermodynamic and physical properties of ferroaluminum (FeAl) and Fe_3_Al by introducing a new embedded atom method (EAM) for a ferroaluminum (FeAl) system that is suitable for atomistic simulations of the kinetic and structural properties of the FeAl system. In 2019, Alizadeh et al.^9^ analyzed a non-vibrational mathematical model to study the mechanical properties of ferroaluminium (FeAl) single crystals derived from molecular dynamics (MD) simulations. They found that the potential energy function of the FeAl alloy influenced stress values. Muralles et al.^10^proposed a modified embedded-atom method to characterize the interatomic potential of ferroaluminum (FeAl), which facilitates molecular dynamics (MD) simulations. They suggested that this modified embedded-atom method could accurately describe the behavior of individual FeAl atoms and compounds under various conditions. Finally, a quasi-static growth model of intermetallic ferroaluminum (FeAl), developed using the Wulf cluster model, was examined in a separate study^11^. In recent years, molecular dynamics simulation has become a crucial tool for understanding the structural characteristics of alloy materials, particularly concerning temperature effects^12,13^. Moreover, the alloying behavior of Al–Mg nanoparticles under different heating conditions has been examined using molecular dynamics simulations, as referenced in^14^. The results demonstrated a strong correlation between heating rates and coalescence kinetics; however, in terms of mechanical properties, the effects were minimal. Jiang et al.^15^ conducted molecular dynamics simulations to investigate the influence of five different heating rates on the sintering of aluminum nanoparticles (diameter: 4–10 nm), primarily focusing on atomic migration. Their findings indicated that as temperature increased, the radial distribution function (RDF) peak gradually diminished, leading to the degradation of the nanoparticles’ crystal structure and their transformation into an amorphous state. In 2023, Arellano-Ramírez et al.^16^ explored the effect of aluminum (Al) nanoparticle size on phase transitions using molecular dynamics simulations. Additionally, Sahni et al.^17^ investigated the atomic-scale process of solid-state sintering in Fe_35_Ni alloy through molecular dynamics simulations. Despite numerous studies on FeAl alloys and Fe- or Al-based materials, most research has focused on macroscale phenomena, such as mechanical properties, as well as microscale aspects, including phase transformations and phase diagrams. However, no study has investigated the effect of heating rates on the melting process of FeAl alloy, particularly concerning its dislocation and structural characteristics at the atomic scale.

In this study, the dislocation behavior and structural characteristics of FeAl under different heating rates are investigated using molecular dynamics simulations. The system temperature is raised beyond the melting point of the FeAl alloy. Additionally, dislocation analysis and phase transitions during the heating process are examined. The primary objective of this study is to enhance the understanding of structural changes in the FeAl alloy at the atomic scale.

## Materials and methods

**Fig. 1:**
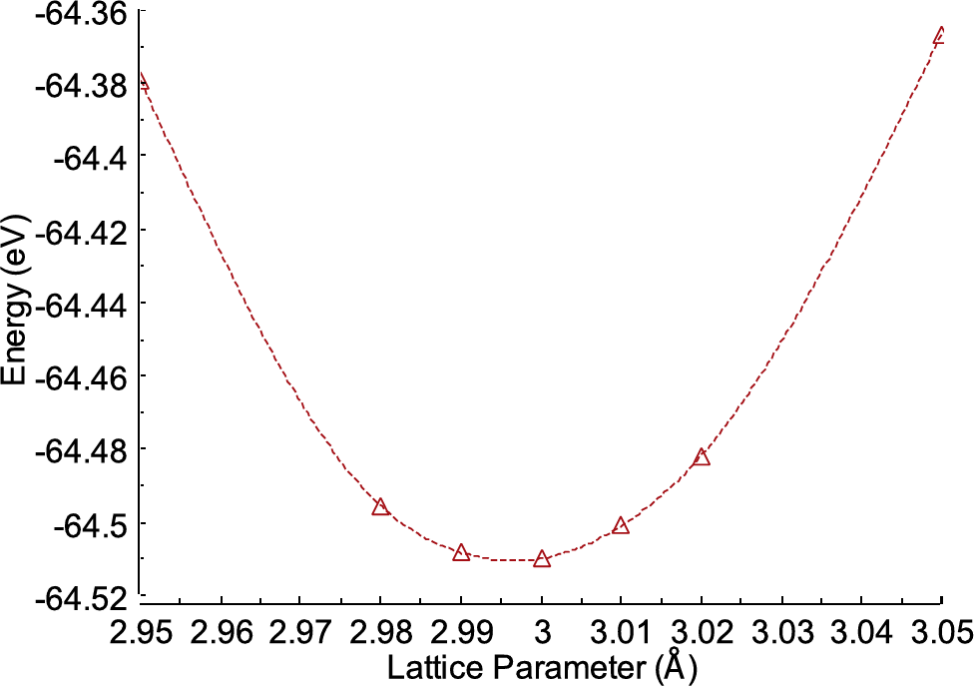
Energy minimum under different lattice parameter of FeAl alloy.

**Fig. 2:**
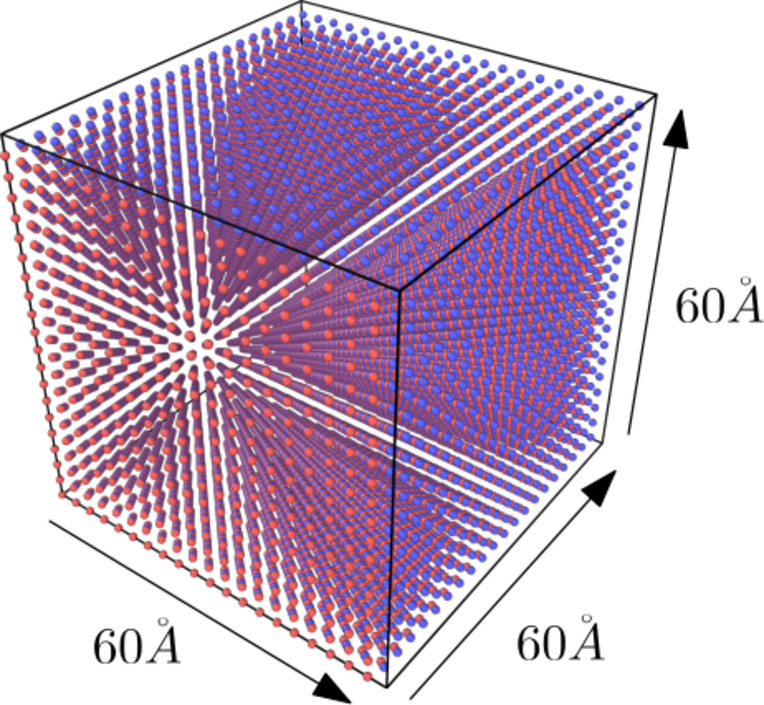
Initial configuration of FeAl alloy with B2 crystal structure. The blue and red balls represent iron and aluminum atoms, respectively.

In this study, a large-scale atomic/molecular massively parallel simulator (LAMMPS) was used to perform molecular dynamics simulations^18^. LAMMPS is a molecular dynamics simulation tool and a classical molecular dynamics code focused on materials modelling. Furthermore, LAMMPS is a parallel particle simulator at the atomic scale, used for solid-state materials such as metals and semiconductors^19,20^, making it relevant to this study. This work used the Embedded Atom Method (EAM),​​ a type of interatomic potential between Fe and Al atoms, as compiled by Zhou et al.^21^. Previous studies have shown that EAM provides a more accurate representation of metallic bonding in materials^22,23^. The initial step in this simulation was to determine the minimum potential performance value or the lattice constant (*a*). The Fe and Al atoms were arranged with parameter values ​​for the x, y, and z axes, which were changed every 0.01 Å, starting from 2.98 Å. The unit cells used were 2 × 2 × 2 cells. Figure 1 shows the lattice parameters with the minimum energy obtained at 3.00 Å. Table 1 compares the lattice constants obtained from the current simulation with experimental lattice constants. After obtaining the value of lattice constant *a*with the minimum energy for the FeAl alloy, the next step was to construct a system with a B2 crystal structure of the FeAl alloy, using a lattice parameter size of 3.00 Å. The FeAl crystal structure was then expanded by 20 × 20 × 20 unit cells along the x, y, and z axes, resulting in a total of 16,000 atoms, comprising 8000 Fe atoms and 8000 Al atoms. Through this expansion, the system size was determined to be 60 × 60 × 60 Å^3^. In our previous study^22^, we compared the pair radial distribution functions from our molecular dynamics simulation with the study by Pham and Giap (2021)^24^and analyzed their behavior across different temperatures. These findings were also compared with experimental data for liquid^25^and amorphous models^26^. The results showed a significant correlation with the experimental data, indicating that the potential suggested by Zhou et al. accurately represents both liquid and amorphous states. Figure 2 shows the initial configuration of the FeAl alloy with a B2 crystal structure.

**Fig. 3:**
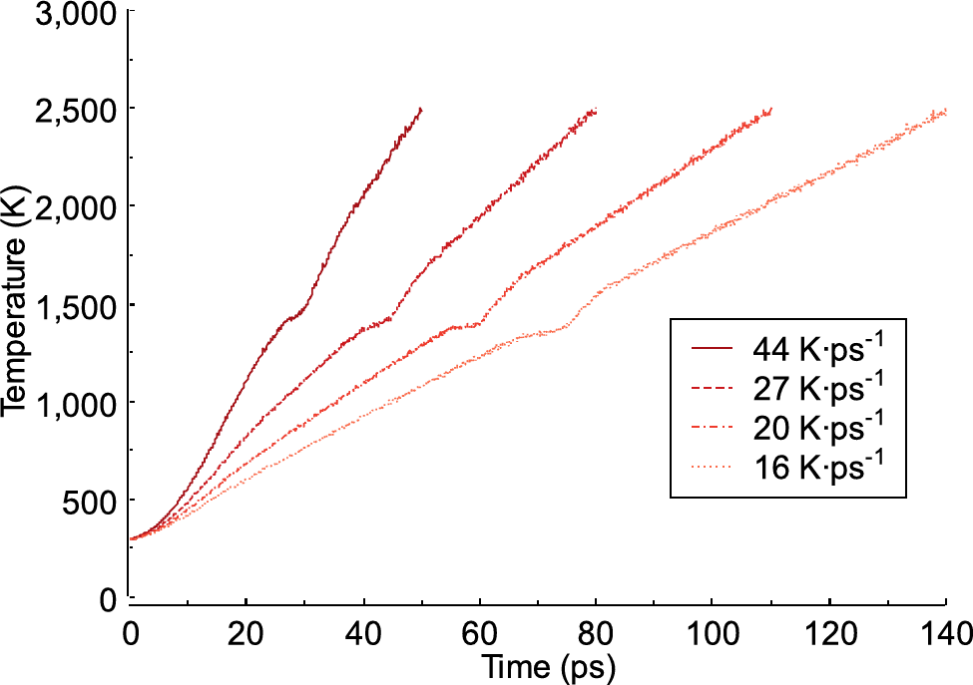
Differences in temperature for heating rates 44, 27, 20, and 16 K ps^−1^.

In this study, periodic boundary conditions were applied to allow atoms leaving one side of the simulation field to be replaced by atoms entering from the opposite side. This approach makes the simulation system appear infinitely large while using a limited number of atoms. Before increasing the temperature in the FeAl system, equilibration was performed for 50 ps at a low temperature of 300 K. To control the temperature and pressure in the system, the isothermal-isobaric (NPT) ensemble scheme, which maintains a fixed pressure (P), a fixed number of atoms (N), and a fixed temperature (T) introduced by Nosé-Hoover^27,28^ was applied. To transform the FeAl crystal structure to a liquid, the temperature was raised from 300 K to 2500 K over 50, ​​80, ​​110, ​​and 140 ps. This temperature was selected to ensure the complete melting of the FeAl alloy system. The heating durations corresponded to heating rates of 44, 27, 20, and 16 K ps^−1^ (Fig. 3). After the heating process, the system was equilibrated at a high temperature of 2500 K for 100 ps. Following equilibration, an (NVT) ensemble, which maintains a fixed number of atoms (N), a fixed cell volume (V), and a fixed temperature (T) was applied for 20 ps, and the results were used for analysis. In this study, data collection for each test was conducted only once. The FeAl structure after the NVT ensemble process was analyzed based on dislocation behaviour, the potential energy, volume changes, radial distribution function g(r), structure factor S(q), and local structure. For visualization, the Ovito^31^program was used, and the structural characteristics were analyzed using Interactive Structure Analysis of Amorphous and Crystalline Systems (I.S.A.A.C.S) software^32^.

**Table 1:** The lattice parameter *a* of feal alloy.

FeAl	Current calculations [Å]	Other calculations [Å]	Experiments [Å]
*a*	3.00	2.995^8^, 2.919^29^	2.90^30^

## Results and discussion

### Effect of heating rate on the potential energy

**Fig. 4:**
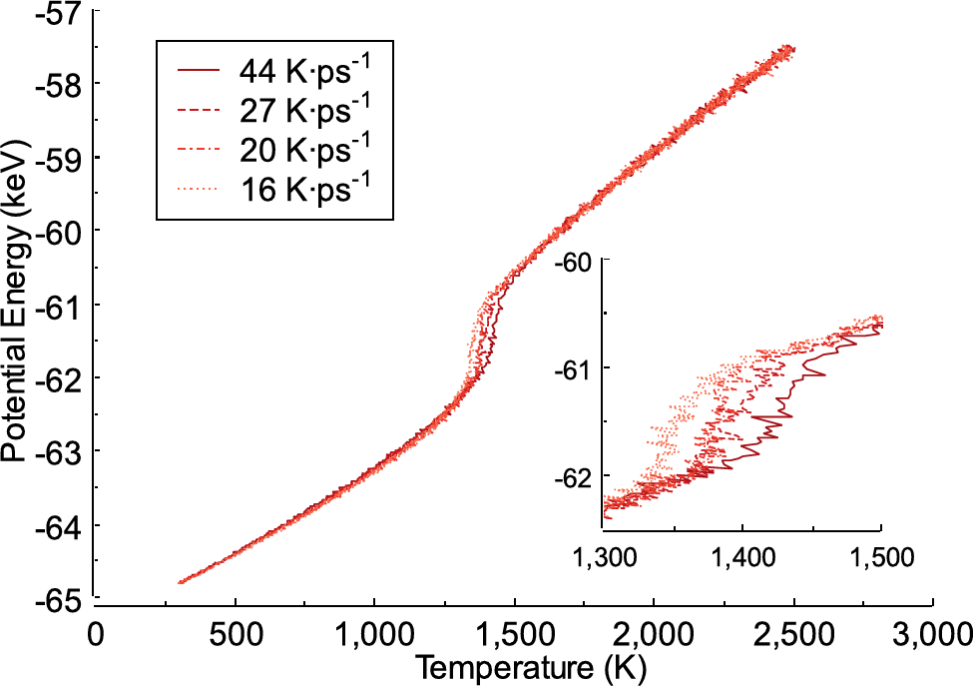
Potential energy of the FeAl alloy generated during the heating process.

In this section, we describe the calculations of the potential energy of the FeAl alloy at different heating rates. At 300 K, the potential energy reaches approximately − 64.81 keV for all heating rates. Subsequently, the potential energy increases with increasing temperature. When the temperature reaches 1350 K, the sum of the atomic potential energies increases with a slight rise in the temperature. Figure 4 shows the potential energy of the FeAl alloy generated during heating. It can be seen from Fig. 4 that the heating rate produces different potential energy values ​​when the temperature reaches 2500 K. For instance, when the heating rate is 44 K ps^−1^, the potential energy in the FeAl alloy system is −57.49 keV. This result differs from that obtained at a heating rate of 27 K ps^−1^, which is −57.51 keV or slightly lower than the potential energy at a heating rate of 44 K ps^−1^. When the heating rates are 20 K ps^−1^ and 16 K ps^−1^, the potential energies at 2500 K are − 57.53 keV and 57.49 keV, respectively.

These findings align with the results as in reference^33^. They found that the energy generated during the heating process of a material increases with increasing temperature. At 2500 K, the potential energy shows only a slight difference across heating rates. However, this difference may also result from the equilibration process, which allows fluctuations in the energy values. The variation in potential energy remains minimal for each heating rate, but a more noticeable difference occurs between 1350 K and 1450 K. Specifically, a slower heating rate of 16 K ps^−1^ leads to an increase in potential energy at around 1350 K, whereas a higher heating rate of 44 K ps^−1^causes this increase at approximately 1450 K. These results align with previous studies, as in references^34,35^, which suggest that this phenomenon corresponds to solid-liquid state transitions^36,37^. Consequently, the nucleation of the liquid phase begins, followed by complete melting^38,39^. This indicates that different heating rates result in distinct nucleation behaviors within the material.

### Effect of heating rate on the volume

**Fig. 5:**
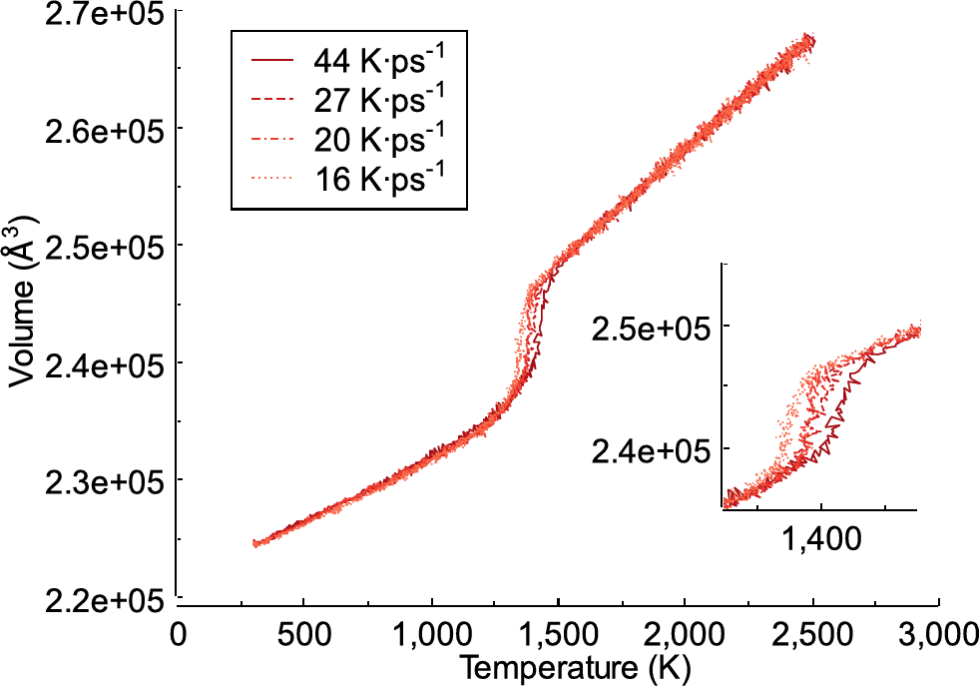
The volume of the FeAl alloy generated during the heating process.

Figure 5 shows the volume change of the FeAl alloy during the heating process at different heating rates. Volume changes during heating are important for the analysis, this is because many applications of this material often involve temperature variations related to thermal expansion. As shown in Fig. 5, as the temperature increases, the volume of the FeAl system also increases. At 300 K, the volumes for all heating rates, 44, 27, 20, and 16 K ps^−1^, are 224486.39 Å^3^. The volume continues to increases as the temperature of the system rise. A phenomenon occurs where the temperature increases slightly, but the volume jumps significantly. This happens at temperatures of 1325, 1350, 1350, and 1400 K for heating rates of 44, 27, 20, and 16 K ps^−1^, respectively. The volume versus temperature trend obtained in this study is consistent with previous reports^34,40^. When the temperature in the system reaches 2500 K, the volume at each heating rate shows different results. This can be seen when the heating rate is 44 K ps^−1^, which produces a volume of 267818.52 Å^3^, compared to the result at a heating rate of 27 K ps^−1^, which is slightly larger at 268029.17 Å^3^. Volume differences also occur at heating rates of 20 K ps^−1^ and 16 K ps^−1^which produce 267466.45 Å^3^ and 267534.11 Å^3^, respectively. This volume difference aligns with previous studies, which show that heating a material causes molecules or atoms to move faster and spread slightly further apart, occupying a larger volume and resulting in a decrease in density^41,42^. The results show that at a faster heating rate, the sudden increase in volume occurs at a higher temperature. This leads to variations in thermal expansion, as reported in previous study^43^. In this study, a faster heating rate of 44 K ps ^−1^ causes a sudden jump in thermal expansion at a slightly higher temperature compared to a slower heating rate of 16 K ps^−1^. This finding is supported by the study conducted by Shor et al.^44^, which demonstrated that a faster heating rate results in a sudden volume increase at a slightly higher temperature than a slower heating rate. This indicates a difference in phase transformation behavior, specifically in the transition from the solid to liquid phase^45^.

Volume changes in the FeAl system cause differences in the density and number density of atoms. When the heating rate is 44 K ps^−1^, the density is 4.111 g/cm^3^, and the number density is 0.060 Atoms/Å^3^. Similarly, at a heating rate of 27 K ps^−1^, the density is 4.114 g/cm^3^ and the number density remains 0.060 Atoms/Å^3^. For heating rates of 20 and 16 K ps^−1^, the densities of the system are 4.114 g/cm^3^ and 4.104 g/cm^3^, respectively, while the number densities are both 0.060 Atoms/Å^3^.

### Effect of heating rate on the radial distribution function

**Fig. 6:**
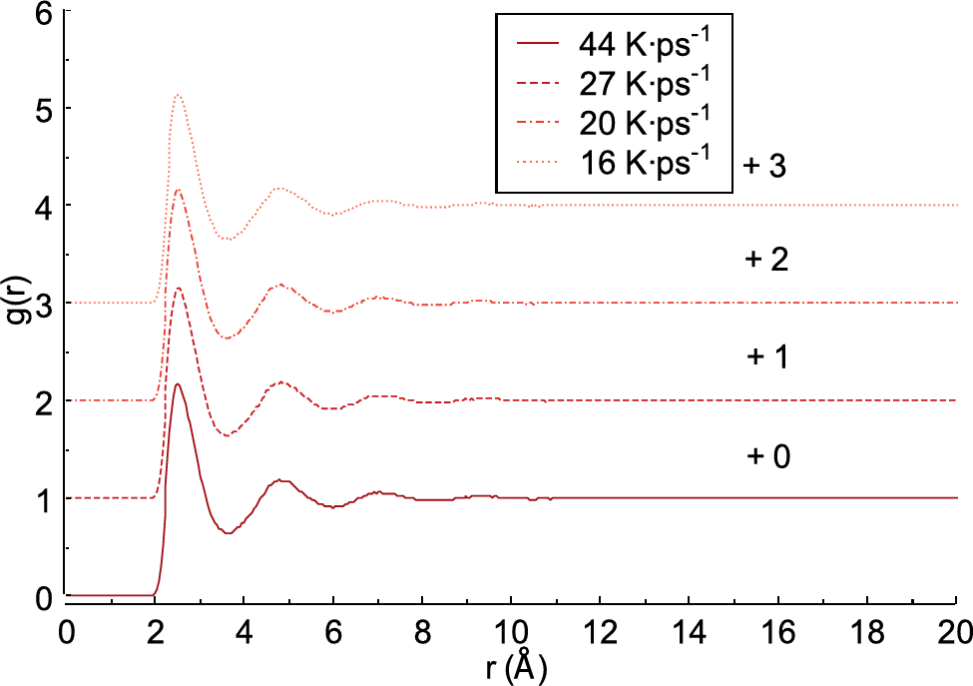
Radial distribution function from X-ray scattering obtained from liquid FeAl alloy.

In this section, the radial distribution function, g(r), of the liquid FeAl alloy is discussed. Two radial distribution function models are considered in this study: X-ray and neutron scattering. Figure 6 shows the radial distribution function obtained from X-ray scattering of the liquid FeAl alloy. As seen in Fig. 6, the radial distribution function g(r) approaches a value of 1 as the distance *r* increases, measured from the center of the reference atom. this can be expressed as follows: g(r) equals zero when *r* is 0 and approaches 1 as *r*tends to infinity^46,47^. The intensity of the first peak in the radial distribution function g(r) varies with the heating rate. When the heating rate is 44 K ps^−1^, the intensity of the first peak is 2.178 and the distance *r* of the first peak is 2.529 Å. Compared to the results from heating rates of 27 and 20 K ps^−1^, the first peak values​​ reach 2.146 and 2.162 at distances *r* = 2.528 Å and 2.528 Å, respectively. This indicates that the intensity of the first peak at 44 K ps^−1^ is 0.032 and 0.016 higher than those at heating rates of 27 and 20 K ps^−1^, respectively. At a heating rate of 16 K ps^−1^, the first peak intensity is 2.135 Å and the *r* value of the first peak is 2.576 Å.

**Fig. 7:**
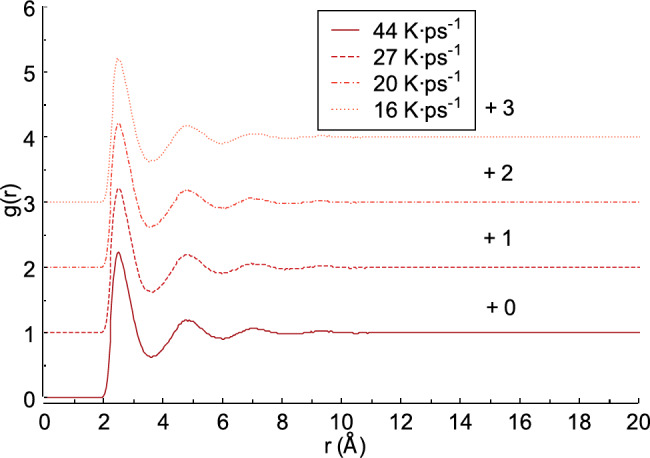
Radial distribution function from neutrons scattering obtained from liquid FeAl alloy.

Figure 7 shows the radial distribution function from the neutron scattering obtained from the liquid FeAl alloy. As seen in Fig. 7, the first peak value varies with different heating rates: 44, 27, 20, and 16 K ps^−1^. For example, when the heating rate is 44 K ps^−1^, the first peak value is 2.232 and the first peak distance *r* is 2.529 Å. At heating rates of 27 and 20 K ps^−1^, the first peak values ​​are 2.204 and 2.216 with *r* distances of 2.528 and 2.528 Å, respectively. A heating rate of 16 K ps^−1^ produces the smallest first peak value of 2.203 with an *r*distance of 2.485 Å. As shown in previous study, the typical radial distribution function (RDF) at high temperatures, such as in the liquid state, consists of several peaks^48^. The overall shape of the RDF closely resembles that of a liquid material, as reported in^49^. However, the shoulder in the second peak often associated with the presence of local icosahedral ordering in the material^24^is not observed in this study. The radial distribution function (RDF) obtained in this work exhibit the following characteristics: the first peak is the most prominent, while the second peak is relatively lower. The first peak represents the average number of particles in the first coordination shell^50^and indicates an inhomogeneous probability distribution of molecular occurrence in the liquid state^51^. Previous studies also have shown that the intensity of the radial distribution g(r) can be influenced by temperature within material^52^and applied pressure^53^.

**Fig. 8:**
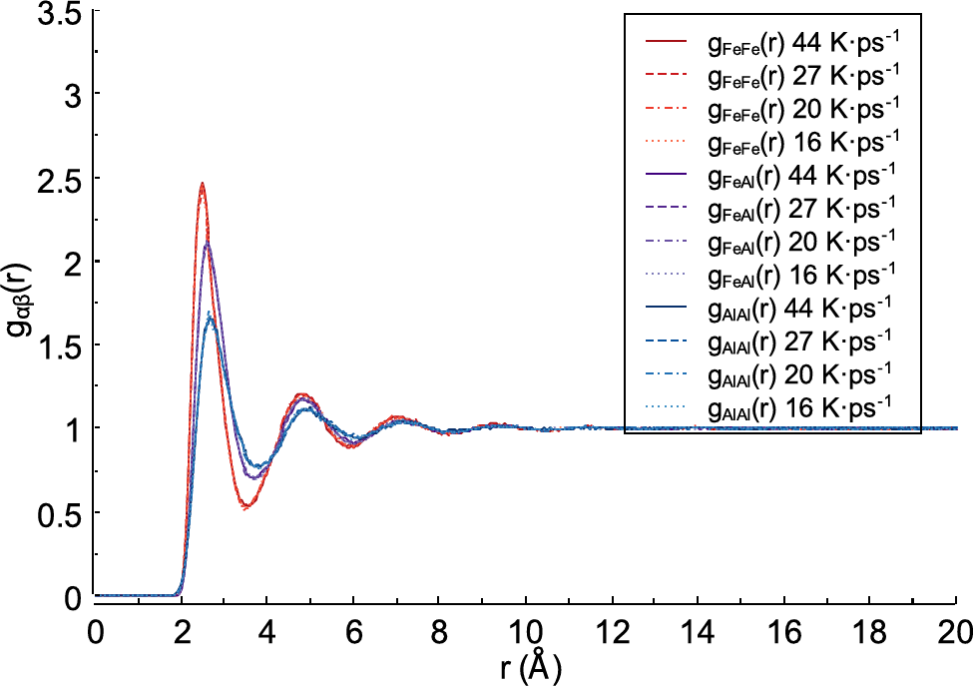
Partial radial distribution functions obtained from liquid FeAl alloy.

Figure 8 shows the partial radial distribution functions obtained for the liquid FeAl alloy. The partial radial distribution function, g_FeFe_(r), has the highest first peak intensity value, followed by g_FeAl_(r) and g_AlAl_(r), for all heating rates. The intensity of the first peak of g_FeFe_(r) at a heating rate of 44 K ps^−1^ was 2.473 Å, and the *r* distance for the first peak is 2.483 Å. This result is higher than the first peak intensities of g_FeAl_(r) and g_AlAl_(r), which are 2.109 and 1.657, with *r* distances of 2.620 Å and 2.665 Å, respectively. At heating rates of 27, 20, and 16 K ps^−1^, the first peak intensities of g_FeFe_(r) are 2.430, 2.452, and 2.458 Å, with *r* distances of 2.482, 2.482, and 2.484 Å, respectively. The first peak intensity values ​​of g_FeAl_(r) and g_AlAl_(r) at heating rates of 27, 20, and 16 K ps^−1^ are 2.127, 2.119, 2.110, 1.665, 1.694, and 1.677, respectively. Meanwhile, the *r* distances for the first peak are as follows: 2.619 Å, 2.619 Å, 2.576 Å, and 2.710 Å, 2.665 Å, 2.667 Å, ​​respectively.

### Effect of heating rate on the structure factors

**Fig. 9:**
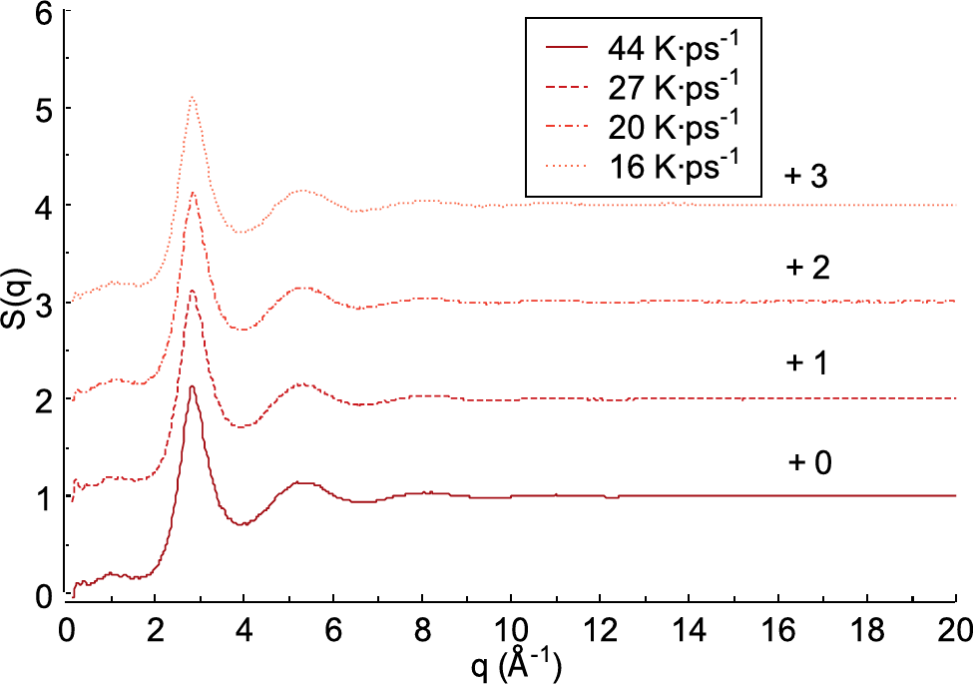
Structure factor from X-ray scattering obtained from liquid FeAl alloy.

The structure factor S(q) is important for analyzing the useful structural characteristics of materials, such as FeAl alloys. Figure 9 illustrates the structure factor obtained from X-ray scattering of the liquid FeAl alloy. As shown in Fig. 9, the heating rate produces different first peak values​​ for the structure factor S(q). Previous studies indicate that the first peak values ​​of the structure factor S(q) are also influenced by the pressure^54,55^. A heating rate of 44 K ps^−1^ yields in the highest first peak value of structure factor S(q) (2.143), with a diffraction vector *q* at the first peak of 2.849 Å^−1^. This differs from the lower heating rates of 27 K ps^−1^ and 20 K ps^−1^where the first peak values of structure factor S(q) are 2.115 and 2.112, with diffraction vectors *q* = 2.849 Å^−1^ and 2.868 Å^−1^, respectively. In this study, the lowest heating rate is 16 K ps^−1^. At this rate, the first peak value of structure factor S(q) is2.101, with a diffraction vector *q* = 2.848 Å^−1^. Among all heating rates, this value is the lowest. The differences in peak values compared to the 44, 27, and 20 K ps^−1^ heating rates are 0.041, 0.014, and 0.011, respectively.

**Fig. 10:**
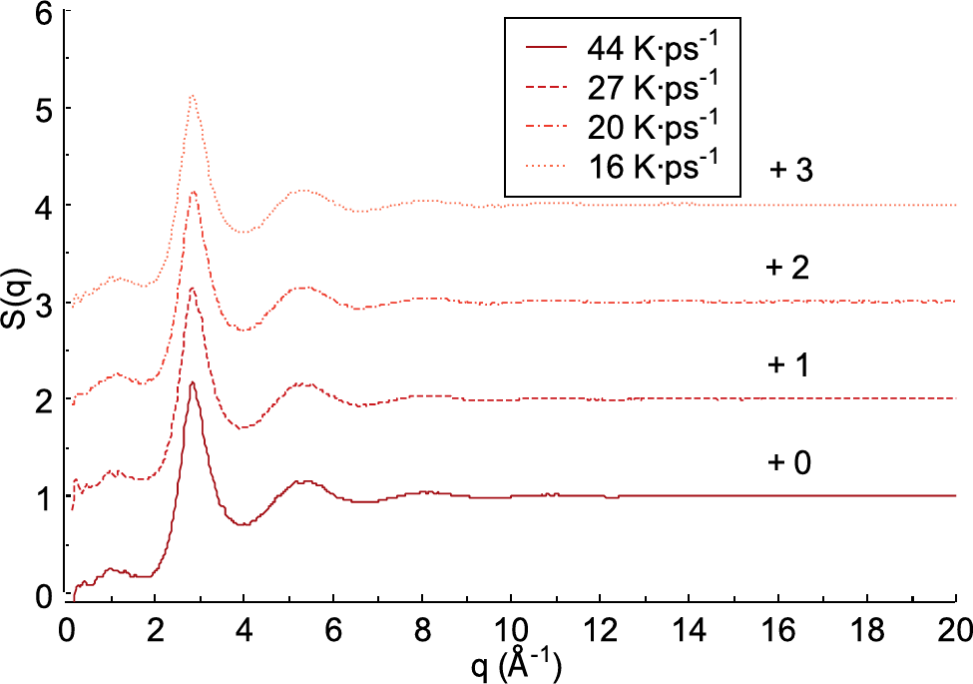
Structure factor from neutrons scattering obtained from liquid FeAl alloy.

In condensed matter physics and crystallography, X-ray and neutron diffraction describe how a material scatters incident radiation, resulting in scattering or interference patterns. These patterns are further interpreted as the structure factor S(q)^56,57^. Figure 10 shows the structure factor S(q) obtained from neutron scattering of the liquid FeAl alloy. The heating rate appears to influence the intensity of the first peak of the structure factor S(q). For example, at a heating rate of 44 K ps^−1^, the intensity of the first peak is 2.170, with a diffraction vector *q* of 2.849 Å^−1^. At a heating rate of 27 K ps^−1^, the intensity of the first peak decreases by 0.026 to 2.144, with the same diffraction vector *q* of 2.849 Å^−1^. This decreasing trend also occurs at heating rates of 20 K ps^−1^ and 16 K ps^−1^. Compared to the intensity at 44 K ps^−1^, the first peak intensity decreases by 0.038 and 0.048, respectively. These two heating rates produce the first peak values of structure factor S(q) at 2.132 and 2.122, with diffraction vectors *q* at the first peaks of 2.868 Å^−1^ and 2.848 Å^−1^, respectively.

### Effect of heating rate on the local structure and dislocation

**Fig. 11:**
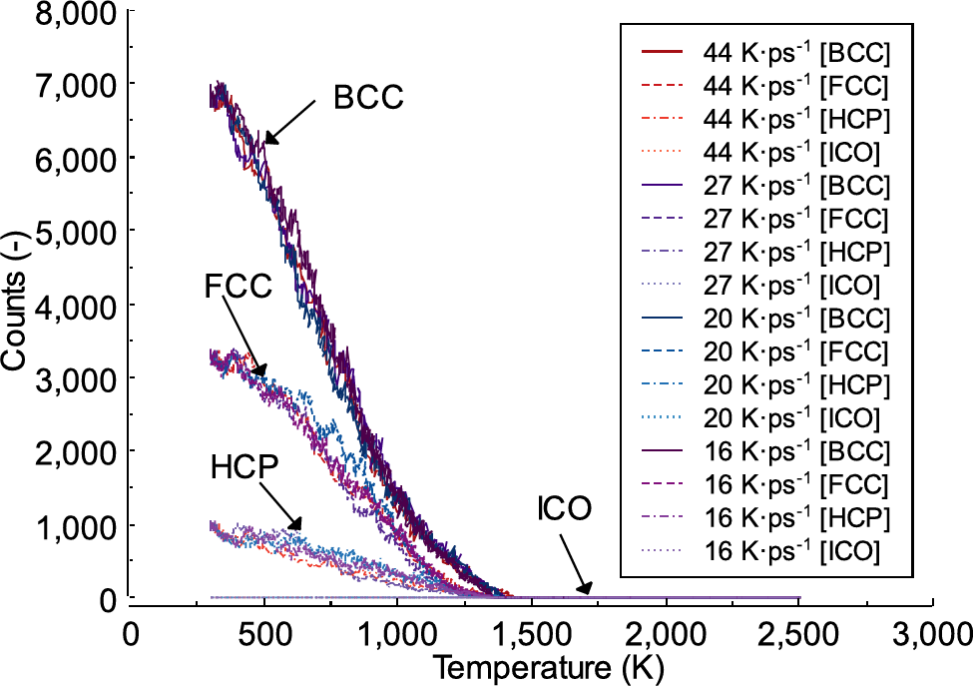
The local structure obtained during the heating process of the FeAl alloy system.

In this section, the distribution of Fe and Al atoms in the local structure is examined. One of the methods used for this analysis is described by^58^. Additionally, the analysis does not differentiate between Fe and Al atoms as local structural units. Figure 11 shows the local structure obtained during the heating of the FeAl alloy system at different heating rates. As observed, the body-centered cubic (bcc) structure is the most prevalent, followed by face-centered cubic (fcc), and hexagonal close-packed (hcp). However, a local icosahedral coordination structure is not found in the FeAl system during the heating process at any heating rate. At 300 K, the local structure bcc is 6858, 3327 for fcc, and 983 for hcp, across all heating rates (44, 27, 20, and 16 K ps^−1^) from a total number of 16,000 atoms. As the system temperature increases, the number of atoms in each local structure decreases. For example, at 1000 K and a heating rate of 44 K ps^−1^, the values are 1192 for bcc, 863 for fcc, and 241 for hcp. For each heating rate, the local structure for bcc varies when the same temperature point is reached, such as at 1000 K. At a heating rate of 27 K ps^−1^, the local structures are 1408 for bcc, 704 for fcc, and 156 for hcp. Compared to heating rates of 20 and 16 K ps^−1^, there is an increase in the total amount of bcc, fcc, and hcp local structures. The increased amounts are as follows: 1483 for bcc, 862 for fcc, and 315 for hcp at 20 K ps^−1^ and 1460 for bcc, 847 for fcc, and 363 for hcp at 16 K ps^−1^. As shown in previous studies, the phase transformation of the crystal structure is not only affected by temperature, but compression in the material also plays a significant role^59,60^.

**Fig. 12:**
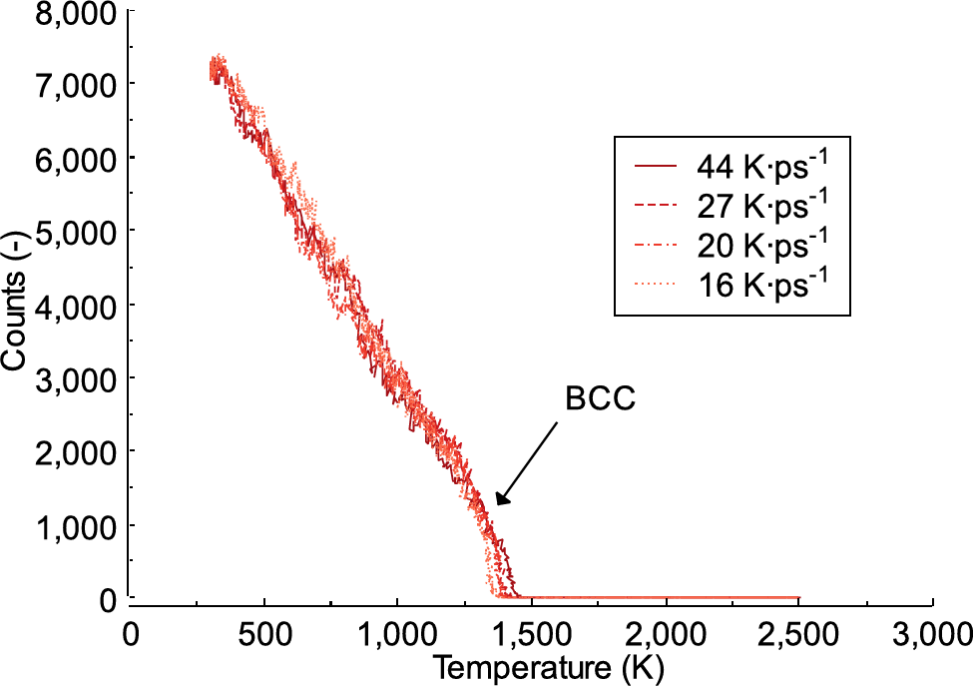
Dislocation analysis of bcc crystal structures at different heating rates in liquid FeAl alloy.

**Fig. 13:**
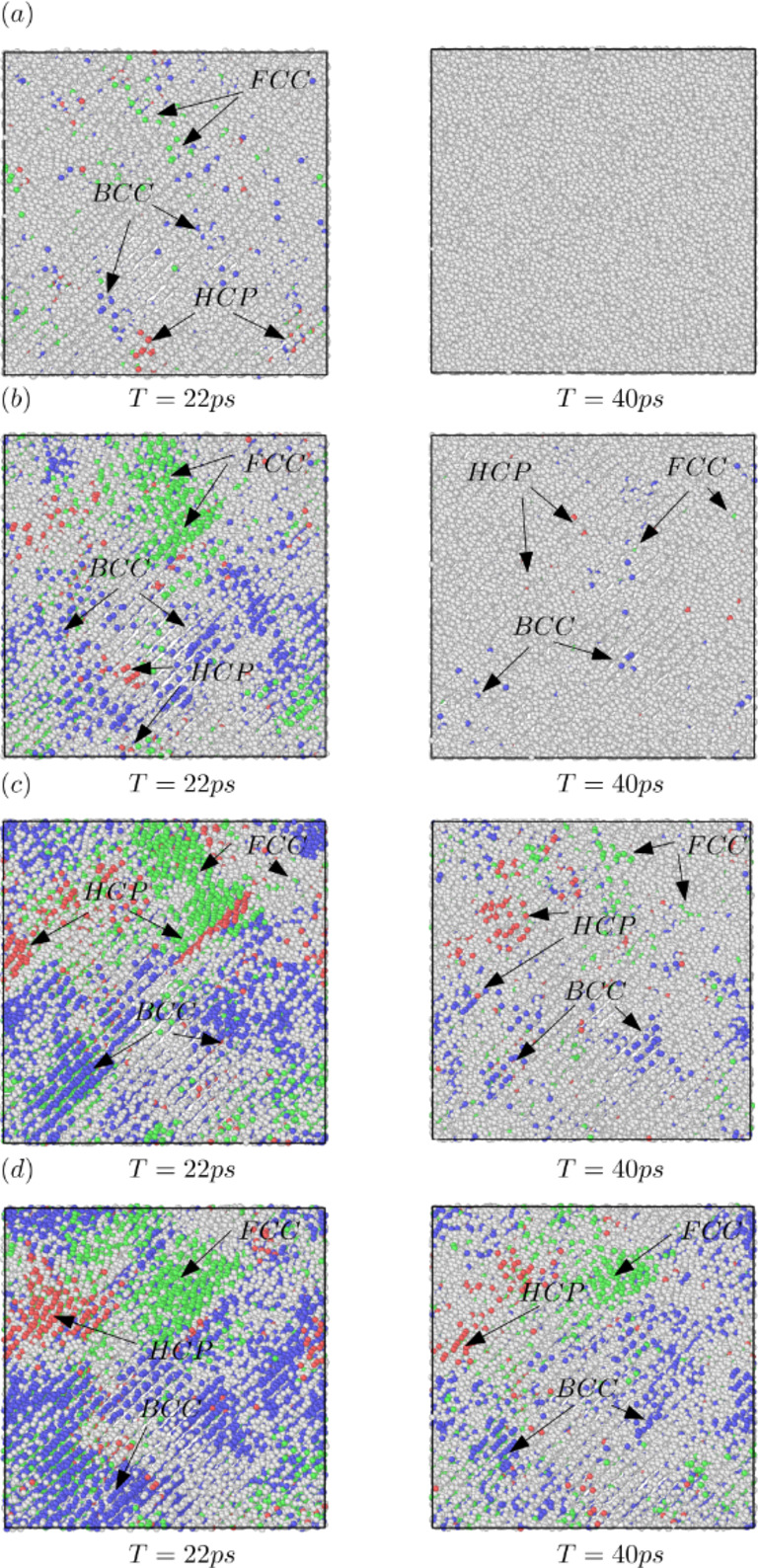
Local structure of liquid FeAl alloy at time (T) = 22 ps and 40 ps. (a) 44 K ps^−1^; (b) 27 K ps^−1^; (c) 20 K ps^−1^; (d) 16 K ps^−1^.

In this study, a slow heating rate of 16 K ps^−1^ produces the lowest number of local structures when the temperature reaches 1400 K, with bcc, fcc, and hcpall equal to 0. However, at the same temperature, a heating rate of 20 K ps^−1^ results in a local structure with bcc and fcc equal to 1 and hcp equal to 2. Higher heating rates, such as 44 K ps^−1^ and 27 K ps^−1^, yield the highest number of local structures. At 1400 K, these values are 47 for bcc, 13 for fcc, and 16 for hcp at 44 K ps^−1^, and 13 for bcc, 5 for fcc, and 2 for hcp at 27 K ps^−1^. In this study, dislocation analysis is performed using the Dislocation Extraction Algorithm (DXA) method. This method quantifies dislocation density in molecular dynamics simulations^61^. The parameter specifies the lattice type of the input crystal, which in this case is bcc. The local crystal orientation is computed to identify the local coordination structure of each atom. For bcc dislocations, a higher heating rate of 44 K ps^−1^ results in dislocations forming at a higher temperature, around 1450 K. In contrast, lower heating rates lead to dislocations forming at lower temperatures; for example, at 16 K ps^−1^, dislocations appear at approximately 1350 K, as shown in Fig. 12. Figure 13shows the local structure of the FeAl alloy at times (T) = 22 ps and 40 ps. As observed, when the time reaches the same value, the heating rate influences the formation of different local structures for bcc, fcc, and hcp. Previous study has shown that the ratio of disordered atoms in the final system is affected by the temperature, indicating that disordered atoms facilitate crystal formation^62^.

In this work, the FeAl structure is initially in the B2 phase, which exhibits the highest local body-centered cubic (bcc) ordering, as shown in Fig. 11. After equilibration, local face-centered cubic (fcc), hexagonal close-packed (hcp), and icosahedral (ico) structures emerge within the material. As the temperature increases, the local structure becomes more disordered due to increased atomic separation. This observation aligns with previous studies^63,64^. Furthermore, the method used to determine local structures may yield different results compared to other approaches, such as the Honeycutt-Andersen method^65^. The findings in this study emphasize the significant impact of heating rates on the structural characteristics and dislocation analysis of FeAl alloys. The results indicate that faster heating rates cause phase transitions to occur at higher temperatures compared to slower heating rates, leading to variations in the distribution of local structures during the transition phase. These insights provide valuable knowledge on the atomic-scale structural evolution of FeAl alloys during heating, particularly for applications that require precise microstructural control. Future studies should incorporate experimental techniques such as X-ray diffraction or neutron diffraction to further analyze the atomic-scale structure of FeAl alloys.

## Conclusions

In this study, dislocation behavior and structural characteristics of FeAl alloys under different heating rates of 44, 27, 20, and 16 K ps^−1^ are examined. Dislocation analysis using the Dislocation Extraction Algorithm (DXA) method shows that faster heating rates lead to higher dislocation temperatures in the bcc crystal structure, while slower heating rates result in lower dislocation temperatures. For instance, a heating rate of 44 K ps^−1^ produces dislocations at approximately 1450 K, whereas a heating rate of 16 K ps^−1^ results in dislocations around 1350 K. Additionally, higher heating rates cause phase transitions to occur at higher temperatures compared to lower heating rates, leading to variations in the distribution of local structures during the transition phase. The results provide a valuable foundation for future experiments, such as determining structural changes and phase transitions in FeAl alloy materials. Future studies should employ experimental methods such as X-ray diffraction or neutron diffraction to analyze the atomic-scale structure of FeAl alloys.

## Data Availability

The datasets generated during and/or analyzed during the current study are available from the corresponding author on reasonable request.
